# Whiplash Maculopathy Following Parachute Injury

**DOI:** 10.18502/jovr.v19i2.7442

**Published:** 2024-06-21

**Authors:** Parveen Sen, Harshit Vaidya

**Affiliations:** ^1^Shri Bhagwan Mahavir, Department of Vitreoretinal Services and Ocular Oncology, Sankara Nethralaya, Chennai, Tamil Nadu, India; ^3^Parveen Sen: https://orcid.org/0000-0003-2256-9380

**Keywords:** Blunt Trauma, Maculopathy, Parachute Injury, Whiplash

## Abstract

**Purpose:**

To report subtle yet important macular changes following a whiplash injury.

**Case Report:**

We report an unusual case of a healthy young male presenting with a three-month history of a drop in vision in both eyes following an accident while crash-landing from a parachute. There was no direct ocular injury. Fundus examination revealed a bilateral well-defined area of retinal pigment epithelium (RPE) alterations over the macula with no other obvious retinal abnormality. Optical coherence tomography (OCT) examination revealed outer retinal layer defects with nearly intact inner retina.

**Conclusion:**

This case highlights the importance of fundus evaluation and reviewing patient's visual symptoms in otherwise inapparent ocular trauma.

##  INTRODUCTION 

Traumatic maculopathy due to inapparent ocular trauma has been noted in cases of “whiplash injury” post vehicular accidents. Various mechanisms have been hypothesized for the trauma causing collateral retinal damage. Ophthalmic complications occur in 25% cases following a whiplash injury. Abrupt acceleration and deceleration forces, as in whiplash injuries can result in varied macular pathologies ranging from isolated outer retinal layer defects to localized retinal detachment.^[1]^ Although whiplash maculopathy is a relatively rare disease entity following trauma, its identification must however not be over-looked as it follows a benign course and warrants only conservative management unlike its graver counterparts like macular hole, retinal detachment, or sub-macular hemorrhage.

**Figure 1:**
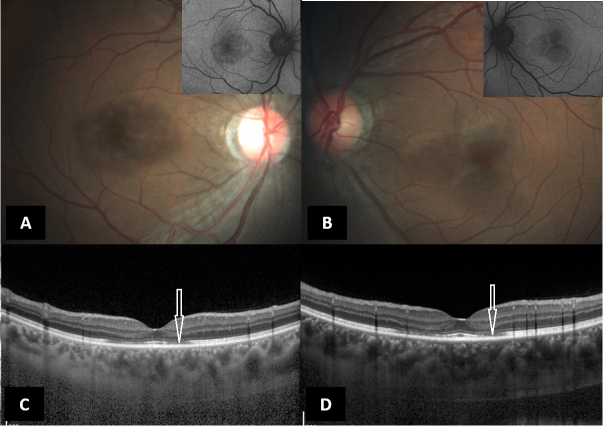
(A & B) Color fundus photograph (Zeiss, Carl Zeiss Meditech, Germany) of the right and left eyes, respectively, showing ass RPE alterations with corresponding hypo autofluorescence (inset). (C & D)Spectral domain optical coherence tomography (Spectralis, Heidelberg Engineering, Germany) of the right and left eyes, respectively, showing parafoveal focal loss and thinning of the ellipsoid zone (white arrows).

**Figure 2:**
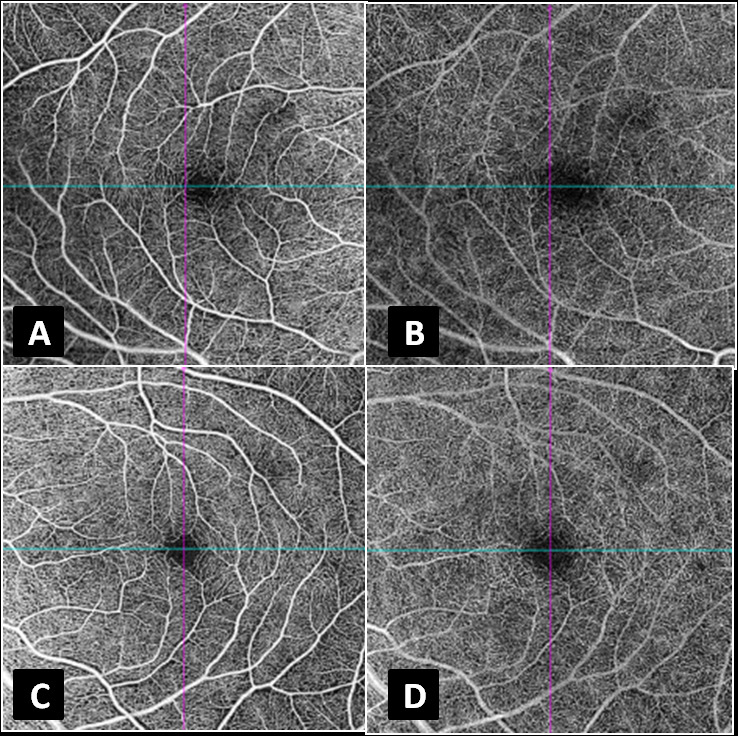
(A & C) Swept source optical coherence tomography–angiography (DRI-Atlantis, Topcon, Oakland, NJ) of the right and left eyes, respectively, segmented at the superficial capillary plexus, showing no evident microcirculatory abnormality. (B & D) Swept source optical coherence tomography–angiography (DRI-Atlantis, Topcon, Oakland, NJ) of the right and left eyes, respectively, segmented at the deep capillary plexus, showing no evident microcirculatory abnormality.

##  CASE REPORT

A 32-year-old male army personnel presented with a three-month history of a drop in vision in both eyes following an accident while landing from a parachute. He sustained no apparent ocular injury, however, he crash-landed onto open fields while sustaining chest injury and minor injuries to the knees as well. His fall also resulted in a reflex hyperextension of the neck. He was wearing a helmet but no protective eye gear. He noticed a diminution in vision three days following the accident. On local consultation, the best corrected visual acuity (BCVA) was recorded as 20/60 in the right eye and 20/20 in the left eye and the optical coherence tomography (OCT) which had been done in another clinic revealed outer retinal defects at the macula. There was no evidence of macular edema and intraretinal hemorrhage or any features of Berlin's edema or Purtscher retinopathy. The patient was empirically on systemic steroids by the local treating ophthalmologist.

At presentation to our clinic, three months after the injury, the patient had similar visual complaints with BCVA of 20/80 in the right eye and 20/30 in the left eye. Anterior segment evaluation of both eyes was essentially normal with normal intraocular pressures and no evidence of angle recession. Fundus examination revealed a symmetrical area of retinal pigment epithelium (RPE) alterations in both eyes with corresponding hypoautofluorescence on fundus autofluorescence. Multicolor and infra-red reflectance images were able to delineate the extent of the pathology corresponding to the hypoautofluorescence images, thereby confirming the outer retinal pathology. Spectral domain OCT (Spectralis, Heidelberg Engineering, Germany) revealed persistent parafoveal focal loss and thinning of the ellipsoid zone (EZ), as was observed in the previous scans without any evidence of posterior vitreous detachment. Minimal outer nuclear layer thinning was observed corresponding to the EZ defects [Figure 1]. Swept source optical coherence tomography angiography (OCTA) revealed no microcirculatory disturbance in the superficial and deep capillary plexuses [Figure 2]. Humphrey visual fields (30-2) of both eyes revealed a paracentral scotoma corresponding to the hypo autofluorescence seen on blue-light fundus autofluorescence (BAF). While the fundus fluorescein angiography and full field electroretinography (ERG) were both normal in both eyes, multifocal ERG revealed reduced foveal and peri-foveal ring responses with normal parafoveal responses in both eyes. The patient was advised to taper systemic steroids and to come for a review after two months. On follow-up, he had a marginal improvement in the symptoms and the BCVA was 20/40 and 20/20 in the right and left eyes, respectively.

##  DISCUSSION 

Retinal involvement following indirect trauma has been classified into three main processes: (1) Retinopathy associated with microcirculatory insufficiency (traumatic retinal angiopathy); (2) Retinopathy associated with systemic coagulopathy as in fat embolization syndrome, pancreatitis, and eclampsia; (3) Maculopathy (whiplash maculopathy) secondary to traumatic posterior vitreous detachment causing photoreceptor damage, with no microcirculatory disturbances.^[2]^ A combination of the three mechanisms may be present in some cases. Although retinal and/or optic nerve sheath hemorrhages have been documented in whiplash injuries, their absence in nonfatal injuries has been noted, as in our case. Retinal changes such as photoreceptor layer disruption, serous macular detachments, sub-RPE fluid, retinoschisis between the outer retinal photoreceptor nuclei and their intact inner and outer segments can occur following whiplash injury.^[3]^ Whiplash maculopathy was first described by Narasaki et al in 1968 and is characterized by a mild reduction in vision (rarely less than 20/30 or more) following a whiplash injury in one or both eyes.^[3]^ Patients generally have an early visual recovery, without any active intervention as reported in literature.^[4]^ However, scotoma is known to persist, which has been arguably attributed to disruption of the outer retinal layers as described by McCannel et al.^[5]^ Kelley et al proposed vitreous traction secondary to traumatic detachment of posterior hyaloid to cause a tiny retinal excavation, seen as a whitish grey circle around the fovea.^[6]^ Capello et al attributed paracentral scotoma seen on visual fields to the defects in the ellipsoid layer, as observed in our case thereby ruling out optic nerve pathology.^[7]^

Our patient characteristically suffered from mild diminution of vision following a whiplash injury secondary to a parachute accident. Although visual recovery was incomplete after three months following the incident, the patient was symptomatically better, and the fundus features and OCT peculiarly displayed stable features. Conservative management was prescribed, as improvement of symptoms and fundus features did not warrant any active intervention.

With increasing popularity of adventure sports, there has been a steady rise in ocular injuries. For instance, subconjunctival and retinal hemorrhages are noted after bungee jumping due to sudden increase in hydrostatic pressure in the retinal circulation.^[8]^ This makes it even more essential to lay down strict safety guidelines pertaining to protective head, neck, and eye gear. Civil aviation requirements pertaining to parachuting provide exhaustive guidelines for the same, however, they fail to endorse the safety measurements regarding appropriate head and eye gear. Although whiplash maculopathy is a transient, non-grievous ocular injury, the whiplash injuries on its own can prove to be fatal. Appropriate measures to prevent such hyperextension injuries, as provided by the extra cushion on the head rest in the four-wheeler vehicles, must extend to the adventure sports as well.

Our report underscores the importance of identification of indirect trauma to the retina following non-ocular injuries and its appropriate characterization to plan further course of management. Multimodal imaging aids in ruling out more severe ocular pathologies following trauma. Patient counselling and reassurance helps allay the fears of this rather non-progressive benign entity.

##  Financial Support and Sponsorship

None.

##  Conflicts of Interest

None.
